# Outcome of traumatic thoracolumbar spine fractures in elderly: A systematic review

**DOI:** 10.1016/j.bas.2024.102775

**Published:** 2024-02-28

**Authors:** J. van Tiel, T. Tan, J. Tee, T.E. Marion, F.C. Öner, J.P.H.J. Rutges

**Affiliations:** a Department of Orthopedic Surgery, University Medical Center Utrecht, Utrecht, the Netherlands; b Department of Neurosurgery, The Alfred Hospital, Melbourne, Victoria, Australia; c National Trauma Research Institute, Melbourne, Victoria, Australia; d Northern Ontario School of Medicine, Thunder Bay, ON, Canada; e Department of Orthopedic Surgery, Erasmus Medical Center, University Medical Center Rotterdam, Rotterdam, the Netherlands

**Keywords:** Spinal trauma, Traumatic vertebral fractures, Thoracolumbar spine, Elderly patient (>65 years), Treatment

## Abstract

**Introduction:**

Adequate guidelines for treatment of people over 65 years, suffering traumatic thoracolumbar spine fractures without neurologic deficit, are currently lacking.

**Research question:**

The aim of this study was to systematically review the available literature regarding the outcome of conservative and surgical treatment of thoracolumbar spinal trauma in elderly patients.

**Material and methods:**

A systematic review according the PRISMA guidelines was performed. Pubmed, Web of Science, EMBASE and the Cochrane Central register were searched until June 2021. Risk of bias of the included studies was evaluated. Clinical and radiological results, as well as complications of conservative or surgical treatment were reviewed.

**Results:**

Six articles were included (one prospective randomized trial, two prospective and three retrospective cohort studies). In these studies conflicting results were observed with regard to pain, radiological results and complications following both conservative and surgical treatment strategies for thoracolumbar spine fractures in elderly.

**Discussion and conclusion:**

Treatment of thoracolumbar fractures in elderly should focus on early mobilization to reduce complications and hospital stay. This may improve functional outcome and prevent worsening of frailty in this vulnerable group of patients. To elucidate the optimal treatment for elderly patient with thoracolumbar fractures, future research should focus on patient specific treatment rather than the mere difference between outcome of surgical and conservative treatment.

## Highlights

• No consensus exists for preferred treatment of traumatic thoracolumbar spine fractures in elderly.
• Fast mobilization after trauma with of without operation decreases bedrest complications.
• The incidence of traumatic thoracolumbar spine fractures in elderly is increasing.

## 1 Introduction

The worldwide demographic situation is rapidly changing. Due to the improvement in health care and improvement of socioeconomic status, life expectancy has considerably increased during the past decades. Consequently, the number of elderly people (which we defined as people aged 65 years and older) is increasing significantly. In 2015 there were 617 million (8.5% of the total population) elderly worldwide and by 2050 the elderly population will have doubled to 1.6 billion (17% of the total population) (https://www.nih.gov/news-events/news-releases/worlds-older-population-grows-dramatically.). In several countries population ageing is far ahead of the worldwide trend. In the Netherlands, for example, already 19% of the general population is aged 65 years or older and in some regions even an exceptional 52% of the local population (https://opendata.cbs.nl/statline/#/CBS/nl/dataset/37296ned/table?dl=6D8D).

On top of the expected global increase of the >65 years population, elderly are also much more active than ten or 20 years ago. The elderly are advised to exercise at least 150 min a week to prevent cardio-vascular disease and diabetes type II (https://www.kenniscentrumsport.nl/ons-aanbod/beweegrichtlijnen/.). Due to the increased knowledge regarding a healthy lifestyle more elderly are involved in competitive sports and are more physically active than in the previous decades. Nordic walking, e-bikes and specialized gym and pool classes have been developed especially for elderly.

Although physical activity and an active lifestyle are in general good for older persons, it also results in higher risks of accidents and physical trauma. Elderly riding an e-bike, for example, are more prone of getting injured than younger e-bike cyclists (Weiss et al., 2018). Moreover, when involved in a trauma, elderly riding an e-bike are more severely injured than elderly riding a traditional bike (Poos et al., 2017). Although most elderly initially recover to their preinjury functional status (Callaway et al., 2007), they usually require more aggressive evaluation and treatment (Dimitriou et al., 2011).

As a result of the higher incidence of traumatic events in physically-active elderly (including motor vehicle accidents, sports and recreational accidents), the relatively poor bone quality and the higher propensity of falling incidents due to frailty, the number of traumatic spinal injuries in this patient population is increasing rapidly (Fassett et al., 2007; Wilson et al., 2020). The treatment of spine fractures in the elderly, however, is a controversial subject (Arul et al., 2019). This is due to the high risk of peri-operative complications, decreased functional recovery at long-term follow-up and increased mortality rate. Both aggressive surgical and more conservative treatment have been suggested, but no consensus exists which treatment is most appropriate (Oner et al., 2017; Bakhsheshian et al., 2014; Yi et al., 2006).

Therefore, the aim of this study is to systematically review the available literature regarding the outcome of conservative and surgical treatment of thoracolumbar spinal trauma in elderly patients.

## 2 Material and Methods

This systematic review was conducted and written according to the Preferred Reporting Items for Systematic Reviews and Meta-Analysis (PRISMA) guidelines (Liberati et al., 2009). This systematic review was registered in the PROSPERO database (PROSPERO 2020 CRD42020138708).

### 2.1 Search strategy and selection criteria

A systematic electronic search of Medline/Pubmed, Web of Science, EMBASE and the Cochrane Central register was performed from inception to June 2021. The search was conducted for spinal fractures, therapy and outcomes combined with a filter for elderly patients. We chose not to include studies including patients with neurological deficit since this is a commonly accepted indication for acute surgical treatment. For all databases the complete search syntax is available in appendix 1. The search strategy, as well as the method of de-duplication of the retrieved articles was published previously by the librarian of the Erasmus Medical Center Rotterdam, Rotterdam, The Netherlands who conducted the search (Bramer et al., 2016, 2018).

Titles and abstracts of the search results were screened independently by two authors (JvT and TT). Inclusion criteria: studies on the treatment and of spinal traumatic injury without neurological deficit in the thoracolumbar spine in elderly patients (aged >65 years). Only studies in English were included. We included the following study designs: meta-analysis, randomized-controlled trials, prospective trials, comparative studies, and large case series (>10 patients). Exclusion criteria were: cervical spine fractures, spontaneous or non-traumatic osteoporotic fractures and pathological fractures (caused by oncological or infectious process). We also excluded the following study designs: case reports, case series <10 patients and review articles.

After applying the selection criteria and selecting suitable articles based on title and abstract, full text articles were read and the bibliography and citing articles of all included studies were screened to identify additional eligible articles which were missed with the initial search.

Any disagreement between the authors selecting the articles for inclusion (JvT and TT) was resolved by consensus.

### 2.2 Data extraction and processing

Extracted data from the included articles was entered into a pre-formatted spreadsheet using Microsoft Excel. Data included author, year of publication, study design, patient demographics, type of treatment (conservative or surgical treatment and type of aforementioned treatment), outcomes (clinical, radiological, and complications) and hospital stay. Data was extracted from full text articles, tables and/or figures by one author (JvT) and accuracy of entered data confirmed by another author (TT).

### 2.3 Assessment of bias

Quality of the included articles was assessed using the criteria published by the Cochrane handbook for systematic review of interventions (Higgins, 2011). The articles were assessed for risk of bias and were rated as ‘‘high risk of bias”, ‘‘low risk of bias” or ‘‘Unclear”. The Newcastle-Ottawa Quality Assessment Scale (NOQAS) was used to assess risk of bias in non-randomized studies (Wells et al.). Any inter-observer disagreement (JvT and TT) was resolved by consensus.

## 3 Results

### 3.1 Search results and included articles

**Fig. 1:**
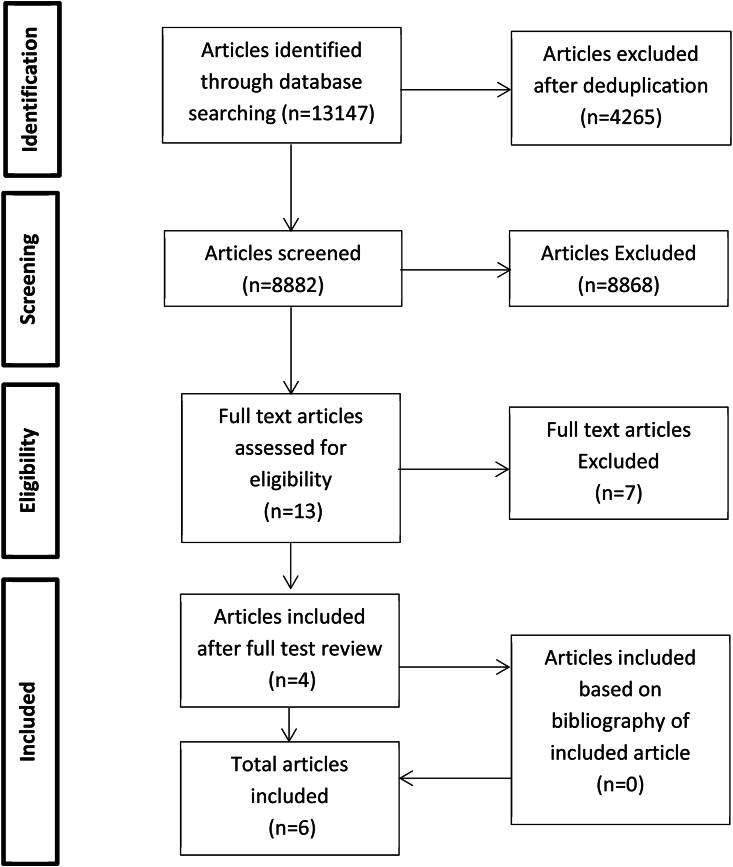
Flowchart.

The search resulted in 13,147 articles, 8882 after de-duplication. The articles were screened on title and abstract. Ten articles were selected for full text review and finally six articles (Cankaya et al., 2015; Eschler et al., 2015; Li et al., 2015; Purvis et al., 2018; Girardo et al., 2020; Kato et al., 2019) were included in our systematic review. These seven articles were excluded for the following reasons: two studies included a combination of post-traumatic and degenerative or spinal cord injuries with and without vertebral fracture cases and unfortunately no separate analyses with only fractures cases was performed (Barbagallo et al., 2017; Lau et al., 2019), two studies included both cervical and thoracolumbar fractures without a separate analysis (Mao et al., 2019; Weerink et al., 2014), one study included patients aged 60 years and older (Kweh et al., 2021), one study included less than 10 cases (Kai et al., 2018) and one study only described the fracture patterns and its levels in diffuse idiopathic skeletal hyperostosis patients (Okada et al., 2019). No additional articles were found based on reference checking of the included articles (Fig. 1).

### 3.2 Study characteristics

From the six included articles, one was a prospective randomized trial with 284 participants (Kato et al., 2019), two were prospective cohort studies (Cankaya et al., 2015; Eschler et al., 2015) with 21 and 15 subjects respectively. The other three included articles were retrospective cohort studies (Girardo et al., Li et al. and Purvis et al.) (Li et al., 2015; Purvis et al., 2018; Girardo et al., 2020) in which 37, 99 and 59,565 subjects were enrolled.

### 3.3 Results of the included articles

Due to small number of included articles and the heterogenous outcomes in these studies, we were only able to give a descriptive overview of the outcomes of the articles. Therefore, a meta analyses was not possible. The demographics and outcomes in the different domains listed below are summarized in Table 1.

**Table 1:** Demographics and results of included studies.

Authors	Number of included patients	Year of publication	Age	Follow-up period	Study population and Type of spinal fracture	Treatment	Clinical outcomes; pain	Radiological outcomes	Complications
Cankaya et al. (2015)	21	2015	71 years (range 60–84 years)	20 months (range 12–26 months)	Traumatic stable compression fractures without posterior tension band injury	Thoracolumbar bracing	Mean VAS pain of 1,62 at final follow-up	In 3 patients increase in local kyphosis in the thoracolumbar spine was observed >30° at final follow-up. This was an incearse of >20° while in all other included patients the increase in kyphosis was only around 10°.	Not studied
Eschler et al. (2015)	15	2015	76 years (range 58–94 years)	10 months (range 5–25 months)	Traumatic AO type A3 fractures	short segment percutaneous pedicle screw placement combined with an expendable cage to restore vertebral body height	Significant reduction in VAS pain from 7.8 to 3.2 at final follow-up	Anterior vertebral body height was significantly improved post-operatively (21.1–23.4 mm), but was lost to 21.5 mm at final follow-up. Local kyphosis was significantly improved post-operatively (9.6–6.0°), but was lost to 8.7° at final follow-up.	Not studied
Girardo et al. (2020)	37	2020	70 years (standard deviation 5 years	34 months (standard deviation 9 months)	Elderly patients over 65 years old with traumatic thoracolumbar osteoporotic fractures type 3–5 according to the German Society for Orthopedics and Trauma^40^	15 patients were treated with sort segment fixation (one level above and below the fractured vertebra) and 21 with a long segment fixation (two levels above and below the fractured vertebra). Screws were augmented if necessary	Significant reduction in VAS pain at final follow-up in all participants	The long segment fixation patients had a significantly better kyphosis at follow-up, while this was nog observed in the short segment group. However, no statistically significant difference in post-operative kyphosis was observed between the groups	In the short segment group, more complications were observed (material failure and proximal junctional kyphosis)
Kato et al. (2019)	284	2019	76 years (standard deviation 5 years	48 weeks	Elderly patients over 65 years old with traumatic thoracolumbar osteoporotic fractures	Thoracolumbar soft of rigid brace treatment for 12 weeks	Less VAS pain in both treatment groups without significant differences between the groups. The brace compliance was not different between the groups.	No difference in anterior compression percentage between the groups	Not studied
Li et al. (2015)	99	2015	71 years (standard deviation 6 years)	Duration of follow-up not mentioned	Elderly polytrauma patients, type of fracture not mentioned	40 treated with kypho- or vertebroplasty, 59 treated conservatively	Not studied	Not studied	3 times more urine tract infections, pneumonia and pressure sores were observed in the conservatively treated group of patients
Purvis et al. (2018)	59,565	2015	83 years (standard deviation 6 years)	Duration of follow-up not mentioned	Patients suffering from traumatic fractures, type of fracture not mentioned	46,962 treated conservatively, 11,116 treated with kypho- or vertebroplasty, 1487 treated operatively	Not studied	Not studied	Patients treated operatively had significantly more (16 versus 8 and 8.1 %) complications compared with patients treated conservatively or with a kypho- or vertebroplasty.

#### 3.3.1 Clinical outcomes; pain

Cankaya et al. reported that in elderly patients (n=21) with radiologically assumed stable compression fractures without posterior tension band injury, the use of a brace is a good conservative intervention for pain reduction (Table 1) (Cankaya et al., 2015). However, no comparison was made with patients not wearing a brace as a control group. In agreement with this study, Kato et al. (2019) showed in 284 patients that both a soft and rigid thoracolumbar brace reduces pain significantly in these patients with a comparable brace compliance independent of the brace type. Eschler et al. (2015) showed that 15 patients with a AO type A3 fractures treated with short segment (one level above and one below the fracture) percutaneous pedicle screw placement above and below the fractured vertebra combined with an expendable cage to restore vertebral body height does also provide satisfactory pain reduction (Table 1). No comparison was made with conservative management or other forms of surgical intervention. In agreement with Echler et al., Girardo et al. (2020) had the same observations in 37 patients and showed that both short segment and long segment (two levels above and below the fractured vertebra) fixation in the thoracolumbar spine resulted in less pain compared to pre-operative pain scores. Functional outcome of elderly patients with thoracolumbar fractures was not reported by the included articles, or not detailed data and analyses was presented.

#### 3.3.2 Radiological outcomes

Although considered as stable fractures, patients with a vertebral compression fracture with intact posterior tension band, Cankaya and colleagues reported progressive kyphotic deformity after conservative treatment in 14% of the patients (3 of 21 patients) (Table 1) (Cankaya et al., 2015). Kato et al. (2019) showed that both, a rigid and soft brace did not prevent anterior compression to occur if thoracolumbar fractures were treated with a brace (114 versus 144 patients in both treatment groups at final follow-up).

Eschler performed a single level percutaneous pedicle screw placement above and below the fractured vertebra combined with an expendable cage to restore vertebral body height in 15 elderly patients with an AO A3 compression fracture to prevent progressive kyphosis. Anterior vertebral body height was restored directly post-operative, however the correction was lost during 6 months of follow-up (Table 1) (Eschler et al., 2015). The middle and posterior vertebral body height did not change from pre to post-operatively and also not at final follow-up. The same pattern as anterior vertebral body height was observed with local kyphosis, e.g. kyphosis was significantly reduced after surgery but at final follow-up it was comparable to pre-operatively. Fracture union was achieved in all cases.

#### 3.3.3 Complications

In the study by Li et al. more complications were observed in elderly polytrauma patients who were treated conservatively for stable vertebral compression fractures compared to patients treated with kypho- or vertebroplasty (59% versus 30% complications) (Table 1) (Li et al., 2015). In 99 patients divided in two groups (40 treated with kypho- or vertebroplasty and 59 treated conservatively), there were more bed rest related complications in the group of patients with a conservative treatment (35% versus 15%) (Table 1). In another study by Purvis and colleagues in which almost 60,000 patients were included and treated conservatively, surgically, or with kypho- or vertebroplasty, the surgical group had the highest number of complications (16% versus 8% in the conservative and kypho- or vertebroplasty group) (Table 1) (Purvis et al., 2018).

### 3.4 Evaluation of bias

Using the NOQAS, three (Cankaya et al. (2015), Eschler et al. (2015) and Li et al. (2015)) out of the four included articles scored poor overall quality (Table 2). This was mainly caused by the lack of the (discretion of) demographic variables between cohorts. Only the article by Purvis et al. (2018) had a good overall quality (Table 2).

**Table 2:** Risk of bias assessment of included observational studies according to the Newcastle-Ottawa Quality Assessment Scale.

First author, Year	Selection				Comparability	Outcome			Quality
	Representativeness of cohort	Selection of non-exposed cohort	Ascertainment of exposure	Outcome of interest	Comparability of cohorts	Assessment of outcome	Adequate duration of follow-up	Adequate follow-up of cohort	
Cankaya et al. (2015)	*	–	*	*	–	*	*	*	Poor
Eschler et al. (2015)	*	–	*	*	–	*	*	*	Poor
Girardo et al. (2020)	*	*	*	*	*	*	*	*	Good
Li et al. (2015)	*	*	*	*	–	*	*	–	Poor
Purvis et al. (2018)	*	*	*	*	*	*	*	–	Good

Good quality: 3 or 4 * in selection domain AND 1 or 2 * in comparability domain AND 2 or 3 * in outcome/exposure domain.

Fair quality: 2 * in selection domain AND 1 or 2 * in comparability domain AND 2 or 3 * in outcome/exposure domain.

Poor quality: 0 or 1 * in selection domain OR 0 * in comparability domain OR 0 or 1 * in outcome/exposure domain.

## 4 Discussion

Currently, no consensus exists on the best treatment strategy for elderly with traumatic thoracolumbar vertebral fractures, therefore the aim of this study was to systematically review the outcome of surgical and conservative treatment. Based on the limited number of included articles and the low level of evidence of these articles we cannot make any strong recommendation on the treatment of thoracolumbar spine fractures in elderly. Nevertheless, some recommendations can be made based on included articles and literature regarding thoracolumbar fractures in elderly.

Short-term follow-up of several weeks of elderly patients with thoracolumbar burst fractures was reported in two studies: patients with intact posterior tension band described by Cankaya et al. and patients with AO A3 fractures described by Eschler et al. (Cankaya et al., 2015; Eschler et al., 2015). All patients had satisfactory results at mid-term follow-up regardless of surgical or conservative treatment. Unfortunately, until now, no studies comparing a conservative and operative treatment for thoracolumbar vertebral fractures in elderly have been performed.

No studies were performed which compared the mid- and long-term outcomes of conservative and surgical treatment of traumatic thoracolumbar vertebral fractures in elderly. Two of the included studies by Li et al. (2015) and Purvis et al. (2018) looked at in-hospital complications of conservatively and operatively managed fractures in elderly. The results of these studies, however, are conflicting since Purvis et al. report more and Li et al. report less complications in the surgical treatment groups. Nevertheless, regardless of the type of treatment, it seems that minimizing the time of mandatory bed rest and early mobilization of the patients may result in fewer complications in geriatric population (Li et al., 2015; Purvis et al., 2018). The most frequently observed complications in both studies are pneumonia, urinary tract infections and pressure sores and were not specifically related to surgical or conservative management strategies. The rate of complications observed in elderly with traumatic vertebral fractures is much higher (ranging from 8 to 60%) regardless of the type of treatment compared to the complication rates (0–25%) in surgically treated younger patients (mean age 41 years) (Gnanenthiran et al., 2012). In agreement with Purvis et al., Gnanenthiran et al. (2012) found less complications in the conservatively treated group than in the operative care group. The fact that elderly patients suffer from more complications in both treatment groups may well be caused by the fact that elderly suffer from more co-morbidities and are more frail than younger patients. To minimize the risk of bedrest-related complications in all patient groups, independent of the age and type of treatment, stimulation of getting out of the hospital bed is of utmost importance for all patients. In addition, the presence of co-morbidities might be considered when making a (shared) decision in which treatment is best for which specific patient. Because of the aforementioned, we can argue that management policy to treat traumatic thoracolumbar fractures in elderly should be similar to more common hip fractures in this age group. This policy can summarized as ‘stabilize‘ the broken hip as soon as possible after trauma to get the patient out of bed and decrease the chance of bedrest complications’ (Brunner et al., 2003). Independent from which treatment is necessary for a vertebral fracture, quick mobilization and less bedrest may then improve outcomes of the chosen treatment.

Eschler et all studied the use of transpedicular expendable cages without cement augmentation to restore height of fractured vertebral bodies (Eschler et al., 2015). Even though they observed a direct post-operative improvement in vertebral body height and decrease in kyphotic deformity, these improvements were lost again at final follow-up 7,5 months after surgery. This outcome is comparable to the results of kyphoplasties and vertebroplasties which restore vertebral height directly post-surgery but this effect is mostly lost during follow-up (Boonen et al., 2011; Wu et al., 2013). Therefore, based on the existing literature, the use of cement and non-cement based augmentation devices in (osteoporotic) compression fractures in elderly is not supported with regard to improving vertebral height, as well as preventing from progressive kyphosis. The use of pedicle screw augmentation with bone cement on the other hand has sown promising results in improving the biomechanical stability of screws in osteoprotic vertebrae when selecting to right patient (Elder et al., 2015; Hoppe et al., 2017).

Although not suitable to restore vertebral body height as well as kyphosis, with regard to quality of life and social implications, the use of kyphoplasties and vertebroplasties might have a beneficial effect in comparison to non-operative treatment for (osteoporotic) vertebral fractures in elderly and therefore might still be considered as a treatment for elderly with a osteoporotic vertebral fracture (Klezl et al., 2012). This way kyphoplasties and vertebroplasties are used in the same way as a hip operation for elderly patients with a hip fracture, for which usually non-operative treatment results in less favorable results in these patients.

This systematic review has several limitations. We were only able to include four studies to answer our research question. All the studies were heterogenic, small number of patients with a relative short follow-up period of maximum 1,5 year after treatment and only 50% of the included studies was prospective. In addition, four studies were of poor quality and/or had a high risk of bias and two studies were rated good quality. Therefore, it was unfortunately not possible to make any strong recommendations based on the current literature.

Because of the limitations of the existing literature and the rapid aging of the world population future research on spine trauma in elderly is urgently needed. This research should take peri- and postoperative risk of morbidity and mortality should into account. Surgical risk assessment could be made using a modified frailty index (mFI). Several studies already showed that frailty is an important predictor of postoperative outcomes following spine surgery (Ali et al., 2016; Flexman et al., 2016). Incorporating the mFI in decision making in traumatic thoracolumbar vertebral fractures in elderly could optimize the (shared) decisions-making process in individual cases. Finally, future research, if surgery is indicated, should also focus on open versus minimal invasive surgery (MIS). Recently two systematic reviews with meta-analyses have been published which advocate to use MIS in thoracolumbar vertebral fractures since there is less blood loss, and less complications, both could be crucial factors in the outcome of frail patients (Pannu et al., 2019; Tian et al., 2018).

In conclusion, treatment of thoracolumbar fractures in elderly should focus on early mobilization to reduce complications and hospital stay. This may improve functional outcome and prevent worsening of frailty in this vulnerable group of patients. To elucidate the optimal treatment for elderly patient with thoracolumbar fractures, future research should focus on patient specific treatment rather than the mere difference between outcome of surgical and conservative treatment.

## Ethics approval

Not applicable.

## Authors' contributions

JvT, TT and JR designed the study. JVT and TT acquired and analyzed the data. All authors (JvT, TT, JT, TM, FO and JR) contributed to interpreting the data, drafting and critically revising the manuscript.

## Funding

Not applicable.

## Consent for publication

Not applicable.

## Availability of data and material

Not applicable.

## Code availability

Not applicable.

## Declaration of competing interest

The authors declare that they have no known competing financial interests or personal relationships that could have appeared to influence the work reported in this paper.
