# Supplementary material for: Outcome of traumatic thoracolumbar spine fractures in elderly: A systematic review
Source: Brain Spine. 2024 Feb 28;4:102775. doi: 10.1016/j.bas.2024.102775 (PMC10951749; doi:10.1016/j.bas.2024.102775)
Supplement: Multimedia component 1 [file mmc1.docx]

**Appendix 1**

Search date 10th of May 2021

Outcome of Traumatic Thoracolumbar Spine Fractures in Elderly: a Systematic Review

| *Database* | *# of refs* | *After de-duplication* |
| --- | --- | --- |
| embase.com | 4885 | 4842 |
| Medline Ovid | 5964 | 3120 |
| Web of science | 2046 | 776 |
| Cochrane CENTRAL | 252 | 144 |
| **Total** | **13147** | **8882** |

**embase.com**

('spine fracture'/mj/de OR 'spinal cord injury'/mj/exp OR 'cervical spine fracture'/mj/de OR 'compression fracture'/mj/de OR (((spin* OR vertebra*) NEAR/3 (fracture* OR injur* OR trauma* OR lesion*))):ti) AND ('therapy'/de OR 'surgery'/de OR 'therapy':lnk OR 'surgery':lnk OR 'spine surgery'/de OR 'conservative treatment'/exp OR 'analgesia'/de OR 'brace'/de OR 'halo brace'/de OR 'cervical collar'/de OR 'spinal brace'/exp OR 'treatment outcome'/de OR 'surgical technique'/de OR 'pedicle screw'/de OR 'fracture immobilization'/de OR 'mobilization'/de OR 'fracture fixation'/de OR (therap* OR treat* OR surg* OR operat* OR nonsurg* OR nonoperat* OR conservative OR bedrest OR analgesia OR brace OR collar OR halo OR immobili* OR immobili* OR fixation OR stabili* OR screw*):ab,ti,kw) AND ('treatment outcome'/de OR 'clinical outcome'/de OR 'critical care outcome'/de OR 'patient-reported outcome'/de OR 'pain assessment'/exp OR 'quality of life'/exp OR 'musculoskeletal function'/de OR 'neuromuscular function'/de OR 'spinal cord function'/de OR 'daily life activity'/exp OR 'complication'/exp OR 'functional assessment'/de OR 'outcome assessment'/de OR 'health status'/de OR 'psychological well-being'/de OR (outcome* OR (pain* NEAR/3 (assess* OR score OR scale OR questionnaire* OR intensit*)) OR (quality NEAR/3 life) OR ((musculoskelet* OR neuromusc* OR spinal-cord* OR assess*) NEAR/3 function*) OR ambulation* OR walking OR (daily NEAR/6 (life OR living) NEAR/6 activit*) OR complication* OR ((health OR functional) NEXT/1 status*) OR well-being OR wellbeing*):ab,ti,kw) AND ('aged'/exp OR 'home for the aged'/exp OR 'nursing home'/de OR 'nursing home patient'/de OR 'aging'/de OR 'geriatrics'/exp OR 'gerontology'/de OR 'geriatric nursing'/de OR 'gerontological research'/de OR 'gerontologist'/de OR 'geriatric care'/exp OR 'geriatric patient'/exp OR 'elderly care'/exp OR 'senescence'/exp OR 'geriatric disorder'/de OR 'geriatric surgery'/de OR 'geriatric assessment'/de OR 'frailty'/de OR 'Longevity'/de OR 'life extension'/de OR 'life expectancy'/de OR (elder* OR ((for-the-aged OR older) NEAR/6 (care OR people OR subject* OR person* OR patient* OR home OR homes OR housing OR adult* OR women OR woman OR female* OR men OR man OR male*)) OR very-old* OR frail* OR old*-age* OR oldest-old* OR ((aged) NEXT/1 (people OR subject* OR person* OR patient* OR population*)) OR senior* OR nursing-home* OR frail* OR aging OR ageing OR geriatric* OR Gerontolog* OR septagenarian* OR octagenarian* OR nonagenarian* OR centenarian* OR supercentenarian* OR senescen* OR Immunosenescen* OR Longevit* OR (life NEXT/1 (exten* OR expectan*)) OR gerontopsych* OR psychogeriat* OR geropsych* OR age-relat* OR ((65 OR 66 OR 67 OR 68 OR 69 OR 70 OR 71 OR 72 OR 73 OR 74 OR 75 OR 76 OR 77 OR 78 OR 79 OR 80 OR 81 OR 82 OR 83 OR 84 OR 85 OR 86 OR 87 OR 88 OR 89 OR 90 OR 91 OR 92 OR 93 OR 94 OR 95 OR 96 OR 97 OR 98 OR 99 OR 100 OR 101 OR 102 OR 103 OR 104 OR 105 OR 106 OR 107 OR 108 OR 109 OR 110 OR 111 OR 112 OR 113 OR 114 OR 115 OR 116 OR 117 OR 118 OR 119 OR 120 OR 121 OR 122) NEXT/1 (year* OR yr OR yrs)) OR ((older-than OR age-of OR aged) NEXT/1 (65 OR 66 OR 67 OR 68 OR 69 OR 70 OR 71 OR 72 OR 73 OR 74 OR 75 OR 76 OR 77 OR 78 OR 79 OR 80 OR 81 OR 82 OR 83 OR 84 OR 85 OR 86 OR 87 OR 88 OR 89 OR 90 OR 91 OR 92 OR 93 OR 94 OR 95 OR 96 OR 97 OR 98 OR 99 OR 100 OR 101 OR 102 OR 103 OR 104 OR 105 OR 106 OR 107 OR 108 OR 109 OR 110 OR 111 OR 112 OR 113 OR 114 OR 115 OR 116 OR 117 OR 118 OR 119 OR 120 OR 121 OR 122))):kw,ab,ti) NOT ([Conference Abstract]/lim OR [Note]/lim OR [Editorial]/lim) AND [english]/lim

**Medline Ovid**

(*Spinal Fractures/ OR *Spinal Cord Injuries/ OR *Fractures, Compression/ OR (((spin* OR vertebra*) ADJ3 (fracture* OR injur* OR trauma* OR lesion*))).ti.) AND (Therapeutics/ OR Surgical Procedures, Operative/ OR therapy.fs. OR surgery.fs. OR exp Conservative Treatment/ OR exp Analgesia/ OR Braces/ OR exp Treatment Outcome/ OR Pedicle Screws/ OR Immobilization/ OR exp Fracture Fixation/ OR (therap* OR treat* OR surg* OR operat* OR nonsurg* OR nonoperat* OR conservative OR bedrest OR analgesia OR brace OR collar OR halo OR immobili* OR immobili* OR fixation OR stabili* OR screw*).ab,ti,kf.) AND (exp Treatment Outcome/ OR Critical Care Outcomes/ OR Patient Reported Outcome Measures/ OR exp Pain Measurement/ OR Quality of Life/ OR Activities of Daily Living/ OR complications.fs. OR exp "Outcome Assessment (Health Care)"/ OR "Patient Outcome Assessment"/ OR Health Status/ OR (outcome* OR (pain* ADJ3 (assess* OR score OR scale OR questionnaire* OR intensit*)) OR (quality ADJ3 life) OR ((musculoskelet* OR neuromusc* OR spinal-cord* OR assess*) ADJ3 function*) OR ambulation* OR walking OR (daily ADJ6 (life OR living) ADJ6 activit*) OR complication* OR ((health OR functional) adj status*) OR well-being OR wellbeing*).ab,ti,kf.) AND (exp Aged/ OR Health Services for the Aged/ OR Homes for the Aged/ OR Housing for the Elderly/ OR Nursing Homes/ OR exp Aging/ OR Geriatrics/ OR Geriatricians/ OR Geriatric Nursing/ OR Geriatric Assessment/ OR Geriatric Psychiatry/ OR Geriatric Dentistry/ OR Dental Care for Aged/ OR Life Expectancy/ OR (elder* OR ((for-the-aged OR older) ADJ6 (care OR people OR subject* OR person* OR patient* OR home OR homes OR housing OR adult* OR women OR woman OR female* OR men OR man OR male*)) OR very-old* OR frail* OR old*-age* OR oldest-old* OR ((aged) ADJ (people OR subject* OR person* OR patient*)) OR senior* OR nursing-home* OR frail* OR aging OR ageing OR geriatric* OR Gerontolog* OR septagenarian* OR octagenarian* OR nonagenarian* OR centenarian* OR supercentenarian* OR senescen* OR Immunosenescen* OR Longevit* OR (life adj (exten* OR expectan*)) OR gerontopsych* OR psychogeriat* OR geropsych* OR age-relat* OR (("65" OR "66" OR "67" OR "68" OR "69" OR "70" OR "71" OR "72" OR "73" OR "74" OR "75" OR "76" OR "77" OR "78" OR "79" OR "80" OR "81" OR "82" OR "83" OR "84" OR "85" OR "86" OR "87" OR "88" OR "89" OR "90" OR "91" OR "92" OR "93" OR "94" OR "95" OR "96" OR "97" OR "98" OR "99" OR "100" OR "101" OR "102" OR "103" OR "104" OR "105" OR "106" OR "107" OR "108" OR "109" OR "110" OR "111" OR "112" OR "113" OR "114" OR "115" OR "116" OR "117" OR "118" OR "119" OR "120" OR "121" OR "122") ADJ (year* OR yr OR yrs)) OR ((older-than OR age-of OR aged) ADJ ("65" OR "66" OR "67" OR "68" OR "69" OR "70" OR "71" OR "72" OR "73" OR "74" OR "75" OR "76" OR "77" OR "78" OR "79" OR "80" OR "81" OR "82" OR "83" OR "84" OR "85" OR "86" OR "87" OR "88" OR "89" OR "90" OR "91" OR "92" OR "93" OR "94" OR "95" OR "96" OR "97" OR "98" OR "99" OR "100" OR "101" OR "102" OR "103" OR "104" OR "105" OR "106" OR "107" OR "108" OR "109" OR "110" OR "111" OR "112" OR "113" OR "114" OR "115" OR "116" OR "117" OR "118" OR "119" OR "120" OR "121" OR "122"))).kf,ab,ti.) NOT (news OR comment* OR editorial* OR congres* OR abstract* OR book* OR chapter* OR dissertation abstract*).pt. AND english.la.

**Cochrane CENTRAL**

((((spin* OR vertebra*) NEAR/3 (fracture* OR injur* OR trauma* OR lesion*))):ti) AND ((therap* OR treat* OR surg* OR operat* OR nonsurg* OR nonoperat* OR conservative OR bedrest OR analgesia OR brace OR collar OR halo OR immobili* OR immobili* OR fixation OR stabili* OR screw*):ab,ti) AND ((outcome* OR (pain* NEAR/3 (assess* OR score OR scale OR questionnaire* OR intensit*)) OR (quality NEAR/3 life) OR ((musculoskelet* OR neuromusc* OR spinal-cord* OR assess*) NEAR/3 function*) OR ambulation* OR walking OR (daily NEAR/6 (life OR living) NEAR/6 activit*) OR complication* OR ((health OR functional) adj status*) OR well-being OR wellbeing*):ab,ti) AND ((elder* OR ((for-the-aged OR older) NEAR/6 (care OR people OR subject* OR person* OR patient* OR home OR homes OR housing OR adult* OR women OR woman OR female* OR men OR man OR male*)) OR very-old* OR frail* OR (old* NEXT/1 age*) OR oldest-old* OR ((aged) NEXT/1 (people OR subject* OR person* OR patient* OR population*)) OR senior* OR nursing-home* OR frail* OR aging OR ageing OR geriatric* OR Gerontolog* OR septagenarian* OR octagenarian* OR nonagenarian* OR centenarian* OR supercentenarian* OR senescen* OR Immunosenescen* OR Longevit* OR (life NEXT/1 (exten* OR expectan*)) OR gerontopsych* OR psychogeriat* OR geropsych* OR age-relat* OR ((65 OR 66 OR 67 OR 68 OR 69 OR 70 OR 71 OR 72 OR 73 OR 74 OR 75 OR 76 OR 77 OR 78 OR 79 OR 80 OR 81 OR 82 OR 83 OR 84 OR 85 OR 86 OR 87 OR 88 OR 89 OR 90 OR 91 OR 92 OR 93 OR 94 OR 95 OR 96 OR 97 OR 98 OR 99 OR 100 OR 101 OR 102 OR 103 OR 104 OR 105 OR 106 OR 107 OR 108 OR 109 OR 110 OR 111 OR 112 OR 113 OR 114 OR 115 OR 116 OR 117 OR 118 OR 119 OR 120 OR 121 OR 122) NEXT/1 (year* OR yr OR yrs)) OR ((older-than OR age-of OR aged) NEXT/1 (65 OR 66 OR 67 OR 68 OR 69 OR 70 OR 71 OR 72 OR 73 OR 74 OR 75 OR 76 OR 77 OR 78 OR 79 OR 80 OR 81 OR 82 OR 83 OR 84 OR 85 OR 86 OR 87 OR 88 OR 89 OR 90 OR 91 OR 92 OR 93 OR 94 OR 95 OR 96 OR 97 OR 98 OR 99 OR 100 OR 101 OR 102 OR 103 OR 104 OR 105 OR 106 OR 107 OR 108 OR 109 OR 110 OR 111 OR 112 OR 113 OR 114 OR 115 OR 116 OR 117 OR 118 OR 119 OR 120 OR 121 OR 122))):ab,ti)

**Web of science**

TI=((((spin* OR vertebra*) NEAR/2 (fracture* OR injur* OR trauma* OR lesion*)))) AND TS=(((therap* OR treat* OR surg* OR operat* OR nonsurg* OR nonoperat* OR conservative OR bedrest OR analgesia OR brace OR collar OR halo OR immobili* OR immobili* OR fixation OR stabili* OR screw*)) AND ((outcome* OR (pain* NEAR/2 (assess* OR score OR scale OR questionnaire* OR intensit*)) OR (quality NEAR/2 life) OR ((musculoskelet* OR neuromusc* OR spinal-cord* OR assess*) NEAR/2 function*) OR ambulation* OR walking OR (daily NEAR/5 (life OR living) NEAR/5 activit*) OR complication* OR ((health OR functional) adj status*) OR well-being OR wellbeing*)) AND ((elder* OR ((for-the-aged OR older) NEAR/5 (care OR people OR subject* OR person* OR patient* OR home OR homes OR housing OR adult* OR women OR woman OR female* OR men OR man OR male*)) OR very-old* OR frail* OR old*-age* OR oldest-old* OR ((aged) NEAR/1 (people OR subject* OR person* OR patient* OR population*)) OR senior* OR nursing-home* OR frail* OR aging OR ageing OR geriatric* OR Gerontolog* OR septagenarian* OR octagenarian* OR nonagenarian* OR centenarian* OR supercentenarian* OR senescen* OR Immunosenescen* OR Longevit* OR (life NEAR/1 (exten* OR expectan*)) OR gerontopsych* OR psychogeriat* OR geropsych* OR age-relat* OR ((65 OR 66 OR 67 OR 68 OR 69 OR 70 OR 71 OR 72 OR 73 OR 74 OR 75 OR 76 OR 77 OR 78 OR 79 OR 80 OR 81 OR 82 OR 83 OR 84 OR 85 OR 86 OR 87 OR 88 OR 89 OR 90 OR 91 OR 92 OR 93 OR 94 OR 95 OR 96 OR 97 OR 98 OR 99 OR 100 OR 101 OR 102 OR 103 OR 104 OR 105 OR 106 OR 107 OR 108 OR 109 OR 110 OR 111 OR 112 OR 113 OR 114 OR 115 OR 116 OR 117 OR 118 OR 119 OR 120 OR 121 OR 122) NEAR/1 (year* OR yr OR yrs)) OR ((older-than OR age-of OR aged) NEAR/1 (65 OR 66 OR 67 OR 68 OR 69 OR 70 OR 71 OR 72 OR 73 OR 74 OR 75 OR 76 OR 77 OR 78 OR 79 OR 80 OR 81 OR 82 OR 83 OR 84 OR 85 OR 86 OR 87 OR 88 OR 89 OR 90 OR 91 OR 92 OR 93 OR 94 OR 95 OR 96 OR 97 OR 98 OR 99 OR 100 OR 101 OR 102 OR 103 OR 104 OR 105 OR 106 OR 107 OR 108 OR 109 OR 110 OR 111 OR 112 OR 113 OR 114 OR 115 OR 116 OR 117 OR 118 OR 119 OR 120 OR 121 OR 122)))) ) AND DT=(article) AND LA=(english)
